# Association of Physical Activity with Phenotypic Age among Populations with Different Breakfast Habits

**DOI:** 10.3390/nu16050575

**Published:** 2024-02-20

**Authors:** Zibo Wu, Jing Li, Yang Xu, Ruirui Guo, Fengdan Wang, Yan Liu, Sizhe Wang, Yibo Dong, Bo Li

**Affiliations:** Department of Epidemiology and Biostatistics, School of Public Health, Jilin University, Xinmin Street No.1163, Changchun 130021, China; wuzb22@mails.jlu.edu.cn (Z.W.); jing_li21@mails.jlu.edu.cn (J.L.); yxu23@mails.jlu.edu.cn (Y.X.); guorr21@mails.jlu.edu.cn (R.G.); wangfd22@mails.jlu.edu.cn (F.W.); yanl22@mails.jlu.edu.cn (Y.L.); szwang22@mails.jlu.edu.cn (S.W.); dongyb22@mails.jlu.edu.cn (Y.D.)

**Keywords:** phenotypic age, physical activity, eating breakfast, inflammation, aging

## Abstract

Background: The global aging situation has reached a serious stage, and healthy lifestyles, like regular physical activity and eating breakfast, could slow the process. Phenotypic age (PhenoAge) is regarded as a novel measure of aging. Therefore, our study aimed to quantify the impact of physical activity and eating breakfast on aging via PhenoAge and phenotypic age acceleration (PhenoAgeAccel). Methods: A total of 3719 adults who participated in the National Health and Nutrition Examination Survey were involved in this study. Physical activity was divided into an active group and an inactive group. According to the number of reported breakfast recalls, eating breakfast was divided into the no recalls group, one recall group, and both recalls group. Sensitivity analysis was performed by stratified analysis. Results: Active physical activity was a protective factor for PhenoAge and PhenoAgeAccel. Compared to the inactive group, the β values of the active group were −8.36 (−10.09, −6.62) for PhenoAge and −1.67 (−2.21, −1.14) for PhenoAgeAccel. The stratified analysis results showed that in the groups reporting breakfast in both recalls, one recall, and no recalls, the β values of the active group were −8.84 (−10.70, −6.98), −8.17 (−12.34, −4.00), and −3.46 (−7.74, 0.82), respectively, compared to the inactive group. Conclusions: Active physical activity was strongly correlated with lower values of PhenoAge and PhenoAgeAccel, but the association was no longer statistically significant when combined with not regularly eating breakfast.

## 1. Introduction

Population aging is an irreversible global trend [1], and the number and proportion of older adults is increasing in almost every country in the world. According to the World Social Report 2023, one-tenth of the world population was made up of older adults in 2021 [2]. This age group is expected to account for one-sixth of the global population by 2050. Aging is one of the major risk factors for most chronic diseases, and rapid population aging could pose a greater public health burden [3]. Therefore, quantifying aging has important implications for the prevention and control of diseases. Chronological age is the traditional indicator for assessing aging, but it does not respond well to the heterogeneity of aging, i.e., different degrees of aging in the population of the same chronological age. Based on traditional clinical chemistry biomarkers [4], a novel aging measure, phenotypic age (PhenoAge), was developed, which can differentiate mortality risk amongst individuals of the same chronological age.

PhenoAge is an epigenetic biomarker closely related to aging developed by Morgan E. Levine based on the National Health and Nutrition Examination Survey (NHANES). It was derived from ten variables (including chronological age) selected from 42 clinical markers and calculated using a formula. The nine biomarkers were albumin, creatinine, glucose, C-reactive protein, lymphocyte percentage, mean cell volume, red blood cell distribution width, alkaline phosphatase, and white blood cell count. This measure is far superior to previous measurements in predicting a variety of aging outcomes, including all-cause mortality, cancer, healthy lifespan, physical function, and Alzheimer’s disease. Although this biomarker was developed using whole blood data, it correlates closely with the age of each type of tissue and cell tested [4]. Previous studies have found that this new biomarker may be a useful tool for facilitating the identification of at-risk individuals, assessing the effectiveness of interventions, and also facilitating research on the underlying biological mechanisms of aging [3].

Following healthy lifestyles could slow down the aging process in daily life [5] and is associated with a slower rate of biological aging [6]. Physical activity is an activity aimed at promoting health, enhancing physical fitness, and enriching life through various sports according to the needs of the body [7]. Biological aging is characterized by the accumulation of molecular and cellular damage [8], leading to structural and functional abnormalities. Regular physical activity can slow down this process [9]. Physical exercise is currently a reliable way to effectively slow down the rate of aging and improve the health of the population.

Besides regular physical activity, breakfast has been described as the most important meal of the day [10] and is already recommended as part of a healthy lifestyle. Skipping breakfast has been reported to be associated with several cardiovascular risk factors, including obesity, diabetes, and coronary heart disease [10,11]. Moreover, several studies have evaluated the contribution of breakfast to daily macronutrient and micronutrient intake, reporting significantly lower intakes of niacin, folate, riboflavin, vitamins C and A, calcium, and iron in breakfast skippers [12,13], which may diminish the body’s antioxidant capacity. Oxidative stress is thought to be detrimental to longevity, and antioxidant substances can attenuate this effect [14]. Thus, eating breakfast could reduce the likelihood of the body being in an inflammatory state and maintain the organism’s younger state, thus exhibiting a lower PhenoAge.

A previous study has indicated that physical activity levels were associated with certain factors of biological aging [15]. Therefore, it may be reasonable to assume that physical activity could affect the rate of biological aging. At the same time, breakfast consumption not only affects aging through inflammation [14], but there is also an association between breakfast consumption and physical activity [16]. However, few studies have focused on the association between physical activity, eating breakfast, and PhenoAge. Therefore, we hypothesized that active physical activity and eating breakfast could be associated with a lower level of PhenoAge and that there may be an interaction between them.

## 2. Methods

### 2.1. Study Population

The NHANES is a set of studies designed to assess the health and nutritional status of adults and children in the United States, providing vital and health statistics for the nation [17]. The study surveys a nationally representative sample of approximately 5000 individuals annually. These individuals are located in counties across the country, 15 of which are visited each year. It utilizes a complex probability sampling design and collects information through standardized interviews, physical examinations, and tests of biological samples. The data for our study were obtained from the NHANES database.

**Figure 1:**
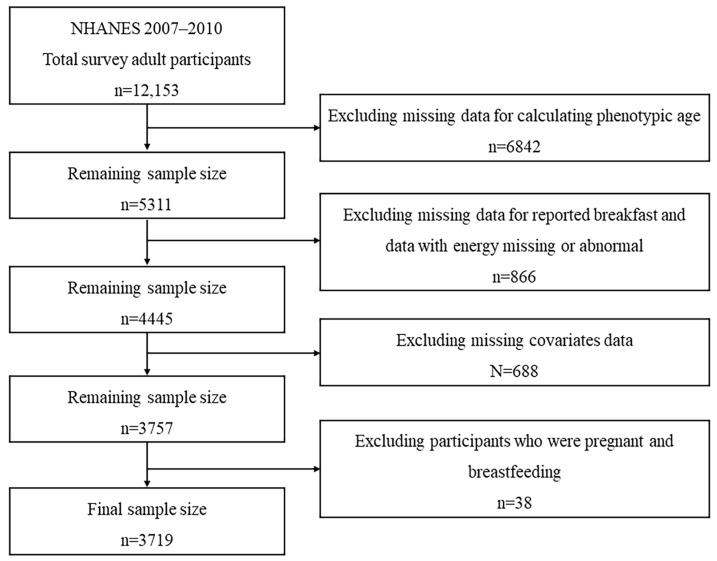
Flowchart of the study design and participants.

Six cycles, from NHANES 1999–2000 through NHANES 2009–2010, surveyed all the indicators needed for PhenoAge calculations and could be used to calculate PhenoAge. However, since the NHANES 2007–2008 cycle, a new calculation method has been used for the calculation of physical activity intensity, and to increase the comparability of the samples and minimize bias, we chose participants from the NHANES 2007–2010 cycles for the study. A total of 12,153 participants aged more than 20 years were included in the 2007–2010 NHANES. We excluded 6842 participants due to the lack of variables required for calculating the PhenoAge. Additionally, 866 participants were excluded for having missing or abnormal data regarding reported breakfast and energy intake (total energy intakes of <500 or >5000 kcal/day for females and <500 or >8000 kcal/day for males were considered to be abnormal). Furthermore, 726 participants with missing values for covariates (BMI, marital status, education status, income status, smoking status, drinking status, and sleep disorder) were also excluded. Participants who were pregnant or breastfeeding were likewise excluded. Ultimately, our study involved 3719 participants. The details are shown in Figure 1.

### 2.2. Data Measurement

#### 2.2.1. Definition of PhenoAge

Levine et al. utilized the Cox penalized regression model along with 10-fold cross-validation to automatically select the most meaningful combination of nine biomarkers (albumin, creatinine, glucose, log[C-reactive protein (CRP)], lymphocyte percentage, mean cell volume, red blood cell distribution width, alkaline phosphatase, and white blood cell count) and chronological age for predicting mortality risk. This selection was made from a total of forty-two biomarkers and chronological age [3,4]. PhenoAge was developed to facilitate the identification of individuals at risk for all-cause mortality, cause-specific mortality, physical functioning, and cognitive performance measures among individuals of similar chronological age [18]. The final equation to calculate PhenoAge is provided below [3]:

$$PhenotypicAge=141.50+\frac{ln\left[-0.00553\times ln\left(1-M\right)\right]}{0.09165}$$

where

$$M=1-exp\left(\frac{-1.51714\times exp(xb)}{0.0076927}\right)$$

$$\begin{array}{ll} xb=-19.907- & 0.0336\times Albumin+0.0095\times Creatinine\\ & +0.1953\times Glucose+0.0954\times \ln\left(CRP\right)\\ & -0.0120\times LymphocytePercent\\ & +0.0268\times MeanCellVolume\\ & +0.3306\times RedCellDistributionWidth\\ & +0.00188\times AlkalinePhosphatase\\ & +0.0554\times WhiteBloodCellCount\\ & +0.0804\times ChronologicalAge \end{array}$$

Taking into account the confounding effects of biological age, we calculated the phenotypic age acceleration (PhenoAgeAccel), defined as the difference between biological age and phenotypic age.

#### 2.2.2. Definition of Physical Activity

Physical activity has been defined as “any bodily movement produced by the contraction of skeletal muscle that increases energy expenditure above a basal level” [19]. The physical activity questionnaire in NHANES was based on the Global Physical Activity Questionnaire (GPAQ) [20], which captures the time spent in various domains of physical activity and by intensity, including vigorous and moderate activity at work, transport activity, and vigorous and moderate activity during leisure time. Metabolic equivalent (MET) values were calculated using conversions recommended by NHANES. Active physical activity was defined as exceeding 599 METs, or more than 149 min of moderate physical activity, or more than 74 min of vigorous physical activity [21].

#### 2.2.3. Eating Breakfast

Data on breakfast were obtained from the NHANES dietary intake data, which were used to estimate the types and amounts of foods and beverages consumed, including all types of water, during the 24-h period prior to the interview (from midnight to midnight). These data also helped to estimate the intake of energy, nutrients, and other food components from those foods and beverages. All subjects in our study participated in two 24-h dietary recall interviews. For each recall in NHANES, the names of meal times and locations were collected. All recalled food or beverage items reported within a specific time frame were assigned the same dietary event designation. Participants who reported eating breakfast, referred to as “breakfast”, “desayuno”, or “almuerzo”, were considered breakfast reporters [22]. Participants who consumed only water with no food intake (resulting in zero energy intake for the day) were required to be excluded [23].

#### 2.2.4. Covariate Assessment

The Dietary Inflammatory Index (DII) is a score derived from the literature, developed to evaluate the inflammatory potential of an individual’s diet [24]. It has been associated with physical activity [25] and breakfast consumption patterns [22], indicating a direct influence on inflammation, where inflammatory diets may contribute to increased inflammation [26]. Given the potential impact of sleep disorders on postprandial glucose control [27] and their causal links to skipping breakfast, lower physical activity levels, and higher DII scores [28,29,30], controlling for these factors was deemed necessary in this study. Participants were categorized into two dietary groups: anti-inflammatory (DII < 0) and pro-inflammatory (DII ≥ 0) [31].

BMI was classified into three groups: under- and healthy weight (BMI < 25.0 kg/m^2^), overweight (25.0 kg/m^2^ ≤ BMI < 30.0 kg/m^2^), and obese (BMI ≥ 30.0 kg/m^2^) [32]. Racial categorization was simplified into non-Hispanic White and all other groups (including Mexican American, other Hispanic, non-Hispanic Black, and other races) [33]. Marital status was defined as either living alone (including widowed, divorced, separated, and never married) or living with someone (including married and living with a partner). Educational attainment was segmented into below high school (including less than 9th grade and grades 9–11, including 12th grade with no diploma), high school graduate/GED or equivalent, and above high school (some college or AA degree and college graduate or above) [34]. Household income was categorized based on the federal poverty level (FPL), adjusting for inflation and family size [35], into ≤130% (low income), >130% to ≤350% (middle income), and >350% (high income) of FPL. Sleep disorder presence was determined by a binary question regarding a doctor-diagnosed sleep disorder.

Smoking status was divided into non-smokers (never had at least 100 cigarettes in their lifetime), former smokers (had at least 100 cigarettes but currently do not smoke), and current smokers (had at least 100 cigarettes and reported smoking in the past 30 days) [36]. NHANES defined one alcohol-based drink as 12 oz. of beer, 4 oz. of wine, or 1.5 oz. of liquor. Drinking status was classified into non-drinkers (did not consume at least 12 alcohol-based drinks in the past year), former drinkers (consumed at least 12 drinks in their lifetime but not in the past year), and current drinkers (consumed at least 12 drinks in the past year and reported frequency of consumption) [36].

### 2.3. Statistical Analysis

All analyses and descriptions were conducted using the complex sampling weights of NHANES. Continuous variables were described using weighted means and standard errors (SEs), and comparisons among groups were made using one-way analysis of variance (ANOVA). Categorical variables were described using unweighted frequencies and weighted percentages, with chi-square tests applied to assess differences between groups. To analyze the effects of physical activity and breakfast consumption on PhenoAge, as well as their interaction effects, general linear models and stratified analysis were employed after adjusting for confounding factors. Furthermore, considering the confounding effect of biological age, the impact of physical activity and breakfast consumption on PhenoAgeAccel was also examined using general linear models and stratified analysis. Forest plots to visualize the results were generated using the “forestplot” package in R. All statistical analyses were performed using IBM SPSS version 26.0 and R version 4.1.2, utilizing the “survey” [37] and “forestplot” [38] packages. A two-sided *p*-value of less than 0.05 was considered statistically significant.

## 3. Results

A total of 3719 participants were involved in this study, including 2940 participants who reported breakfast in both recalls, 584 participants who reported breakfast in one recall, and 195 participants who reported breakfast in no recalls. The mean PhenoAge across all participants was 42.09 years. Specifically, it was 44.48 years for participants who reported breakfast in both recalls, 35.57 years for those who reported breakfast in one recall, and 30.63 years for participants who reported breakfast in no recalls. Statistically significant differences were observed across the breakfast reporting groups in terms of PhenoAge, gender, race, education status, marital status, income status, smoking status, drinking status, and DII (*p* < 0.05). However, no statistical differences were found in physical activity and PhenoAgeAccel among the breakfast groups (*p* > 0.05). The details are shown in Table 1. In terms of physical activity, there were 2211 participants in the active group and 1508 in the inactive group. The mean PhenoAge was 38.39 years for the active group, in contrast to 48.91 years for the inactive group. The details are shown in Table S1.

Table 2 presents the outcomes of the general linear model analyses assessing the effects of physical activity and breakfast reporting frequency on PhenoAge across three different models. Consistently across all models, participants who were more physically active and those who reported fewer breakfast instances exhibited lower PhenoAge levels. After controlling for gender, race, education level, marital status, income level, BMI, DII, energy intake, smoking status, drinking status, and sleep disorder, the β coefficient for the active group, in comparison to the inactive group, was −8.36 (−10.09, −6.62). For breakfast reporting, the β coefficients for reporting breakfast on one recall and no recalls, as compared to reporting breakfast on both recalls, were −6.02 (−8.03, −4.01) and −11.40 (−15.53, −7.26), respectively, with *p* < 0.05 indicating statistical significance. Results from Table S2 for the general linear model analysis of physical activity and reported breakfast on PhenoAgeAccel revealed that physical activity continued to serve as a protective factor against PhenoAgeAccel (*p* < 0.001), with a β coefficient for the active group of −1.67 (−2.21, −1.14) compared to the inactive group. However, the effect of reported breakfast frequency on PhenoAgeAccel was not statistically significant (*p* > 0.05).

**Figure 2:**
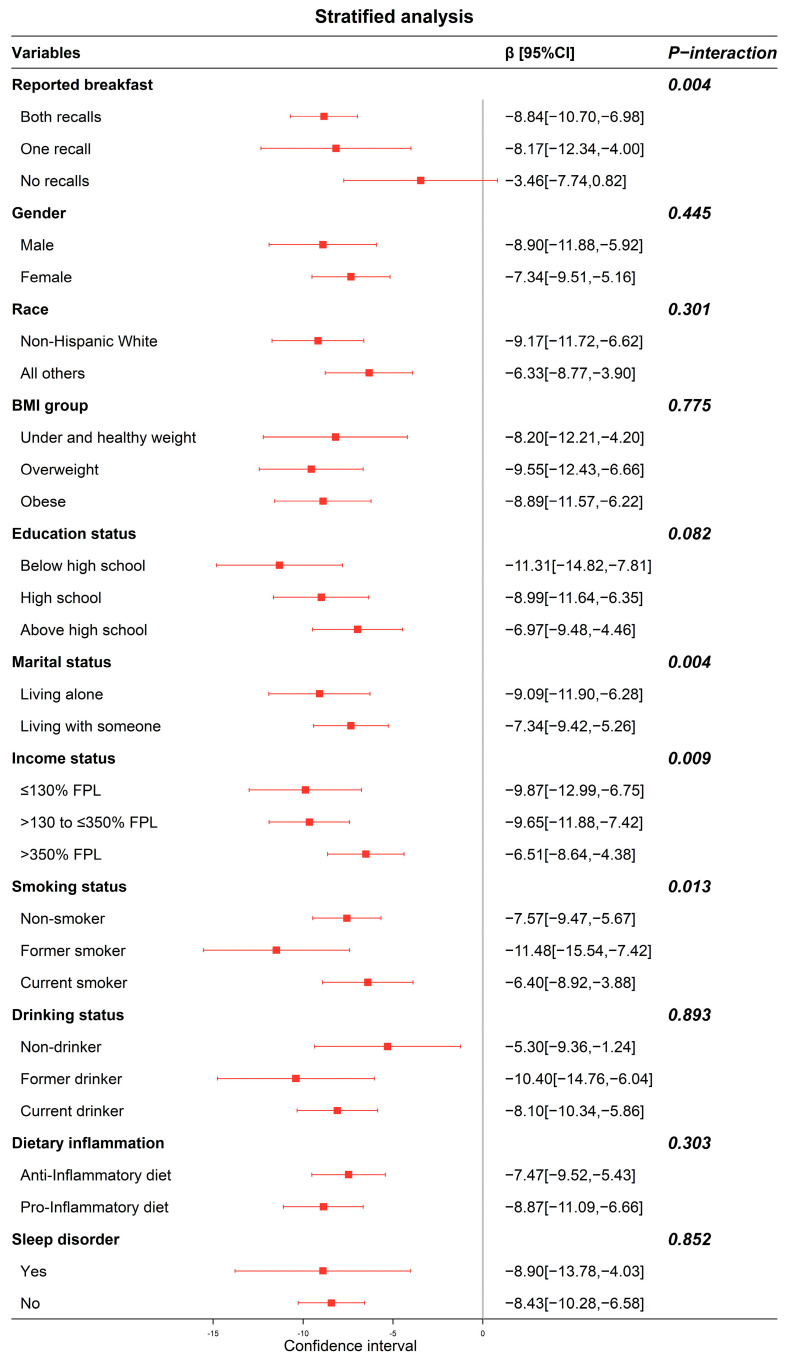
Forest plot of stratified analyses of the associations between physical activity and PhenoAge. Note: the control group was physically inactive. Adjusted for reported breakfast, gender, race, education status, marital status, income status, BMI, DII, energy intake, smoking status, drinking status, and sleep disorder. Bold indicates the value of the *P*-interaction for the grouping variable.

In subsequent analyses, we explored the multiplicative interactions and conducted stratified analyses to examine the association between physical activity and PhenoAge across different subgroups, with the findings presented in Figure 2. Significant interactions were observed between physical activity and reported breakfast, marital status, income status, and smoking status in influencing PhenoAge, with *p*-values for interaction at 0.004, 0.004, 0.009, and 0.013, respectively. Within the subgroups of reported breakfast in both recalls, one recall, and no recalls, the β values for the active group were −8.84 (−10.70, −6.98), −8.17 (−12.34, −4.00), and −3.46 (−7.74, 0.82), respectively, in comparison to the inactive group.

Figure S1 illustrates the results from multiplicative interaction analyses and stratified analyses assessing the relationship between physical activity and PhenoAgeAccel across different subgroups. The interaction between physical activity and reported breakfast on PhenoAgeAccel yielded a *p*-value of 0.109. For participants who reported breakfast in both recalls, one recall, and no recalls, the β values for the active group were −1.63 (−2.24, −1.03), −2.71 (−4.22, −1.19), and −0.36 (−1.17, 1.88), respectively, when compared to the inactive group.

## 4. Discussion

In this study, we explored the association between physical activity, eating breakfast, and PhenoAge. We found that active physical activity was negatively associated with PhenoAge and PhenoAgeAccel across the total population. Additionally, there was an interaction between physical activity and eating breakfast on PhenoAge. Further stratified analyses revealed that, compared to being physically inactive, engaging in active physical activity serves as a protective factor against PhenoAge and PhenoAgeAccel among individuals who reported consuming breakfast in both or one of the recalls. However, this protective effect was not statistically significant among those who reported breakfast in no recalls. To our knowledge, this study is the first to focus on the interaction between physical activity and eating breakfast in relation to PhenoAge.

Considering that physical activity and healthy lifestyles, including eating breakfast, are known to be effective in preventing age-related diseases, it is reasonable to propose that these lifestyle factors may also play an active role in delaying the aging process [39]. The aging process is thought to be associated with persistent systemic inflammation [40]. A previous study based on NHANES data [41] demonstrated a negative correlation between leisure time physical activity in the elderly and markers of inflammation, such as CRP, segmented neutrophil count, and alkaline phosphatase, thereby illustrating the impact of leisure time physical activity on the dose and intensity response to inflammation. This negative association between physical activity and inflammatory factors was further supported by the findings of Tessa J. Parsons [42]. Additionally, this conclusion was reinforced by the results of an animal study, in which mice given access to a running wheel for six weeks exhibited a reduction in the number of all leukocyte subpopulations in their blood [43]. Moreover, physical activity is known to influence, and be influenced by, circadian clocks [44], which in turn affect aging through various complex pathways [45]. Therefore, the impact of physical activity on PhenoAge may be mediated through mechanisms involving inflammation and circadian rhythms.

Moreover, we observed that skipping breakfast undermines the beneficial effects of physical activity. The trend of skipping breakfast is increasing in modern lifestyles [46], with this behavior being especially prevalent among younger individuals [47]. Evidence suggests that the prolonged fasting associated with skipping breakfast can heighten the activity of inflammatory vesicles and the inflammatory response of peripheral leukocytes [46], as well as increase concentrations of inflammation biomarkers, including CRP. Skipping breakfast might impair glycemic control and modify the inflammatory response to meals, potentially leading to an uptick in neutrophil numbers and the production of pro-inflammatory cytokines [48,49]. Consequently, skipping breakfast could negate the positive effects of physical activity by elevating the body’s inflammation levels, thus accelerating aging effects. Furthermore, akin to physical activity, regular breakfast habits might also confer health benefits by enhancing circadian rhythms and, in turn, decelerating aging [50,51]. Although skipping breakfast diminishes it, active physical activity can still contribute to lowering PhenoAge, albeit not reaching statistical significance. This suggests that physical activity may partially offset the adverse effects of skipping breakfast on aging.

The relationship between eating breakfast and PhenoAge diverged from our initial expectations, a discrepancy we attribute to varying levels of chronological age across the three populations that reported breakfast consumption, given that chronological age is the predominant factor influencing PhenoAge. It appears that younger demographics are more inclined to skip breakfast [52]. Taking this confounding factor into account, we also computed PhenoAgeAccel as an alternative outcome variable and observed that the correlation between breakfast consumption and PhenoAgeAccel ceased to be statistically significant. Therefore, the link between breakfast consumption and PhenoAge warrants further investigation. Additionally, our research indicated no statistical difference in physical activity levels among the diverse breakfast reporting groups, suggesting that breakfast habits do not directly influence physical activity levels. Nevertheless, an interaction was detected between physical activity and breakfast habits in relation to aging, indicating that breakfast habits may modulate the relationship between physical activity and aging through alternative mechanisms, such as inflammation [40] and circadian rhythms [45], as previously discussed. Given these insights, we recommend that American adults cultivate active exercise routines to lower PhenoAge and decelerate the aging process, a benefit that could be augmented by consistently consuming breakfast.

Hormesis is defined as the adaptive response of cells and organisms to moderate stresses, which can be caused by exercise, diet, and environmental exposures [53]. Hormesis could enhance resilience to normal aging, thereby preventing disease and improving the health of the population [54]. Previous studies have found that humans have an exercise-associated hormesis curve, where lack of exercise increases the risk of infection, moderate exercise strengthens the immune system, and excessive exercise leads to damaging oxidative stress [55,56]. Similar to exercise, diet also has this hormesis curve. According to hormesis theory, repeated exposure to mild-intensity stress triggers beneficial effects on different organs and systems [57,58]. We can achieve delayed aging and healthy aging based on the theory. Therefore, future research should focus on the association of aging with hormesis and the potential factors that influence it.

### 4.1. Study Strengths

This study has several strengths. Among its strengths, it is noteworthy for being the first to focus on the interaction between physical activity and eating breakfast in relation to PhenoAge, a recently developed composite indicator for predicting aging. Additionally, our study provides evidence supporting the role of eating breakfast and engaging in physical activity in reducing PhenoAge and delaying aging. Lastly, it is based on NHANES, a nationally representative survey.

### 4.2. Study Weaknesses

Regarding its weaknesses, the study’s cross-sectional design means that its conclusions regarding causal inferences may be less reliable. Additionally, since NHANES data pertain to Americans, our results may not be applicable to populations in other countries. The study aimed to explore lifestyle factors affecting PhenoAge, relying on self-reported responses, which could impact the results.

### 4.3. Future Research Directions

Based on our study, we suggest that future research should focus on the mechanisms of how breakfast affects the association between physical activity and aging, with attention to the confounding role of biological age. In addition, further studies should also examine the association between aging and hormesis, including potential influencing factors. Specifically, the role of healthy lifestyles, such as physical activity and regular breakfast consumption, in modulating hormesis and delaying aging should be a focal point.

## 5. Conclusions

Active physical activity was strongly correlated with lower levels of PhenoAge and PhenoAgeAccel. However, this association was not statistically significant when combined with irregular breakfast consumption. It is recommended to enhance the aging process positively by maintaining regular physical activity and consistent breakfast consumption.

## Figures and Tables

**Table 1:** Characteristics of participants reporting breakfast in both, one, or no recalls ((Mean (SE))/N (%)).

Characteristics	Total (N = 3719)	Reported Breakfast in			F/χ2	*p*
		Both Recalls (N = 2940)	One Recall (N = 584)	No Recalls (N = 195)		
PhenoAge, years	42.09(0.65)	44.48(0.80)	35.57(1.07)	30.63(1.78)	74.548	<0.001
PhenoAgeAccel, years	−5.53(0.17)	−5.64(0.21)	−5.32(0.28)	−4.87(0.56)	2.676	0.069
Physical activity					1.137	0.566
Inactive	1508(35.1)	1205(35.5)	228(33.8)	75(34.0)		
Active	2211(64.9)	1735(64.5)	356(66.2)	120(66.0)		
Age, years	47.62(0.57)	50.12(0.71)	40.89(0.91)	35.50(1.58)	119.795	<0.001
Gender					21.978	<0.001
Male	1806(48.3)	1383(46.4)	299(52.4)	124(59.6)		
Female	1913(51.7)	1557(53.6)	285(47.6)	71(40.4)		
Race					19.291	<0.001
Non-Hispanic White	1969(73.7)	1608(75.5)	262(66.3)	99(71.6)		
All others	1750(26.3)	1332(24.5)	322(33.7)	96(28.4)		
BMI, kg/m^2^	28.76(0.13)	28.70(0.17)	28.89(0.33)	29.19(0.54)	1.357	0.258
BMI group					6.540	0.162
Under and healthy weight	1046(30.6)	817(30.4)	177(32.9)	52(26.1)		
Overweight	1291(33.4)	1045(34.2)	176(29.0)	70(35.8)		
Obese	1382(36.0)	1078(35.4)	231(38.1)	73(38.1)		
Education status					16.130	0.003
Below high school	1003(18.2)	793(17.8)	158(19.8)	52(18.9)		
High school	880(23.4)	667(21.9)	146(25.2)	67(36.1)		
Above high school	1836(58.4)	1480(60.3)	280(55.0)	76(45.0)		
Marital status					37.107	<0.001
Living alone	1442(36.9)	1069(34.0)	270(43.8)	103(53.5)		
Living with someone	2277(63.1)	1871(66.0)	314(56.2)	92(46.5)		
Income status					54.803	<0.001
≤130% FPL	1122(20.5)	809(17.4)	230(30.0)	83(31.5)		
>130 to ≤350% FPL	1437(35.6)	1158(36.3)	203(31.7)	76(38.0)		
>350% FPL	1160(43.9)	973(46.3)	151(38.3)	36(30.5)		
Smoking status					104.174	<0.001
Non-smoker	2001(54.3)	1620(55.7)	292(49.6)	89(50.4)		
Former smoker	989(26.8)	834(29.1)	128(22.5)	27(10.3)		
Current smoker	729(18.9)	486(15.2)	164(27.9)	79(39.3)		
Drinking status					17.223	0.002
Non-drinker	480(10.3)	411(11.4)	56(7.1)	13(5.4)		
Former drinker	552(12.3)	439(13.0)	88(10.6)	25(9.1)		
Current drinker	2687(77.4)	2090(75.6)	440(82.3)	157(85.5)		
DII	−0.10(0.09)	−0.35(0.07)	0.38(0.15)	1.74(0.29)	101.671	<0.001
Dietary inflammation					119.751	<0.001
Anti-Inflammatory	1791(53.8)	1543(58.9)	209(43.5)	39(20.2)		
Pro-Inflammatory	1928(46.2)	1397(41.1)	375(56.5)	156(79.8)		
Energy, kcal	2119.99(19.50)	2116.63(19.18)	2203.81(68.86)	1937.37(67.81)	1.009	0.365
Sleep disorder					1.039	0.595
Yes	275(7.1)	224(7.4)	38(5.6)	13(6.9)		
No	3444(92.9)	2716(92.6)	546(94.4)	182(93.1)		

**Table 2:** General linear models for physical activity and reported breakfast on PhenoAge.

	Model 1 ^a^		Model 2 ^b^		Model 3 ^c^	
	β [95%CI]	*p*	β [95%CI]	*p*	β [95%CI]	*p*
Physical activity (reference = Inactive)	−10.39 [−12.45, −8.33]	<0.001	−10.03 [−12.13, −7.92]	<0.001	−8.36 [−10.09, −6.62]	<0.001
Reported breakfast (reference = Both recalls)	—	—	—	—	—	—
One recall	−8.73 [−11.17, −6.29]	<0.001	−7.98 [−10.50, −5.47]	<0.001	−6.02 [−8.03, −4.01]	<0.001
No recalls	−13.69 [−17.98, −9.41]	<0.001	−13.72 [−18.01, −9.42]	<0.001	−11.40 [−15.53, −7.26]	<0.001

^a^ Model 1 without adjustments. ^b^ Model 2 additionally adjusted for gender (male, female), race (non-Hispanic, all others), education status (below high school, high school, above high school), marital status (living alone, living with someone), income status (≤130%FPL, >130 to ≤350%FPL, >350%FPL). ^c^ Model 3 additionally adjusted for BMI (kg/m^2^), DII, energy intake (kcal), smoking status (non-smoker, former smoker, current smoker), drinking status (non-drinker, former drinker, current drinker), and sleep disorder (yes, no).

## Data Availability

The data that support the findings of this study are openly available at https://www.cdc.gov/nchs/nhanes/ (accessed on 18 October 2023). Information from NHANES is made available through an extensive series of publications and articles in scientific and technical journals. For data users and researchers throughout the world, survey data are available on the internet and easy-to-use CD-ROMs.

## Supplementary Materials

The following supporting information can be downloaded at: https://www.mdpi.com/article/10.3390/nu16050575/s1.
