# Supplementary material for: Association of Physical Activity with Phenotypic Age among Populations with Different Breakfast Habits
Source: Nutrients. 2024 Feb 20;16(5):575. doi: 10.3390/nu16050575 (PMC10934488; doi:10.3390/nu16050575)
Supplement: Supplementary file 1 [file nutrients-16-00575-s001.zip › nutrients-2853372-supplementary.pdf]

Table S1 Characteristics of participants with active and inactive physical activity ((Mean (SE))/N (%)).

**Characteristics**	**Total (N=3719)**	**Physical activity**		z/χ^2	***P***
		**Active (N=2211)**	**Inactive (N=1508)**		
PhenoAge, years	42.09(0.65)	38.39(0.69)	48.91(0.88)	-17.378	<0.001
PhenoAgeAccel, years	-5.53(0.17)	-6.43 (0.17)	-3.88(0.30)	-8.726	<0.001
Reported breakfast				1.137	0.566
Both recalls	2940(76.6)	1735(76.2)	1205(77.5)		
One recall	584(17.0)	356(17.3)	228(16.3)		
No recalls	195(6.4)	120(6.5)	75(6.2)		
Age, years	47.62(0.57)	44.82(0.65)	52.79(0.69)	-16.245	<0.001
Gender				99.544	<0.001
Male	1806(48.3)	1223(54.6)	583(36.6)		
Female	1913(51.7)	988(45.4)	925(63.4)		
Race				3.870	0.049
Non-Hispanic White	1969(73.7)	1200(74.8)	769(71.7)		
All others	1750(26.3)	1011(25.2)	739(28.3)		
BMI, kg/m^2	28.76(0.13)	28.06(0.17)	30.06(0.17)	-7.314	<0.001
BMI group				50.428	<0.001
Under and healthy weight	1046(30.6)	672(32.9)	374(26.1)		

Overweight	1291(33.4)	820(36.1)	471(28.5)		
Obese	1382(36.0)	719(31.0)	663(45.4)		
Education status				78.196	<0.001
Below high school	1003(18.2)	498(14.9)	505(24.4)		
High school	880(23.4)	497(21.7)	383(26.5)		
Above high school	1836(58.4)	1216(63.4)	620(49.1)		
Marital status				8.403	0.004
Living alone	1442(36.9)	815(37.0)	627(36.7)		
Living with someone	2277(63.1)	1396(63.0)	881(63.3)		
Income status				46.280	<0.001
≤130% FPL	1124(20.5)	625(18.7)	497(23.7)		
>130 to ≤350% FPL	1437(35.6)	802(33.6)	635(39.4)		
>350% FPL	1160(43.9)	784(47.7)	376(36.9)		
Smoking status				1.367	0.505
Non-smoker	2001(54.3)	1190(54.9)	811(53.3)		
Former smoker	989(26.8)	576(26.5)	413(27.3)		
Current smoker	729(18.9)	445(18.6)	284(19.4)		
Drinking status				70.595	<0.001
Non-drinker	480(10.3)	219(8.2)	261(14.2)		
Former drinker	552(12.3)	285(10.8)	267(15.2)		
Current drinker	2687(77.4)	1707(81.0)	980(70.6)		

DII	-0.10(0.09)	-0.36(0.10)	0.39(0.11)	-10.999	<0.001
Dietary inflammation				86.600	<0.001
Anti-Inflammatory	1791(53.8)	1204(59.2)	587(43.8)		
Pro-Inflammatory	1928(46.2)	1007(40.8)	921(56.2)		
Energy, kcal	2119.99(19.50)	2209.37(21.40)	1954.82(31.26)	11.741	<0.001
Sleep disorder				13.223	<0.001
Yes	275(7.1)	135(5.3)	140(10.5)		
No	3444(92.9)	2076(94.7)	1368(89.5)		

Table S2 General linear models for physical activity and reported breakfast on PhenoAgeAccel.

	**Model 1**[a]		**Model 2**[b]		**Model 3**[c]	
	**β [95%CI]**	***P***	**β [95%CI]**	***P***	**β [95%CI]**	***P***
Physical activity (reference=Inactive)	-2.56[-3.17,-1.95]	<0.001	-2.55[-3.17,-1.94]	<0.001	-1.67[-2.21,-1.14]	<0.001
Reported breakfast (reference=Both recalls)	—	—	—	—	—	—
One recall	0.37[-0.32,1.05]	0.281	-0.01[-0.63,0.62]	0.982	-0.29[-0.88,0.29]	0.313
No recalls	0.81[-0.50,2.11]	0.215	0.05[-1.22,1.31]	0.939	-0.87[-2.03,0.29]	0.136

[a] Model 1 without adjustments. [b] Model 2 additionally adjusted for gender (male, female), race (non-Hispanic, all others), education status (below high school, high school, above high school), marital status (living alone, living with someone), income status (≤130%FPL, >130 to ≤350%FPL, >350%FPL). [c] Model 3 additionally adjusted for BMI (kg/m2), DII, energy intake(kcal), smoking status (non-smoker, former smoker, current smoker), drinking status (non-drinker, former drinker, current drinker), and sleep disorder (yes, no).

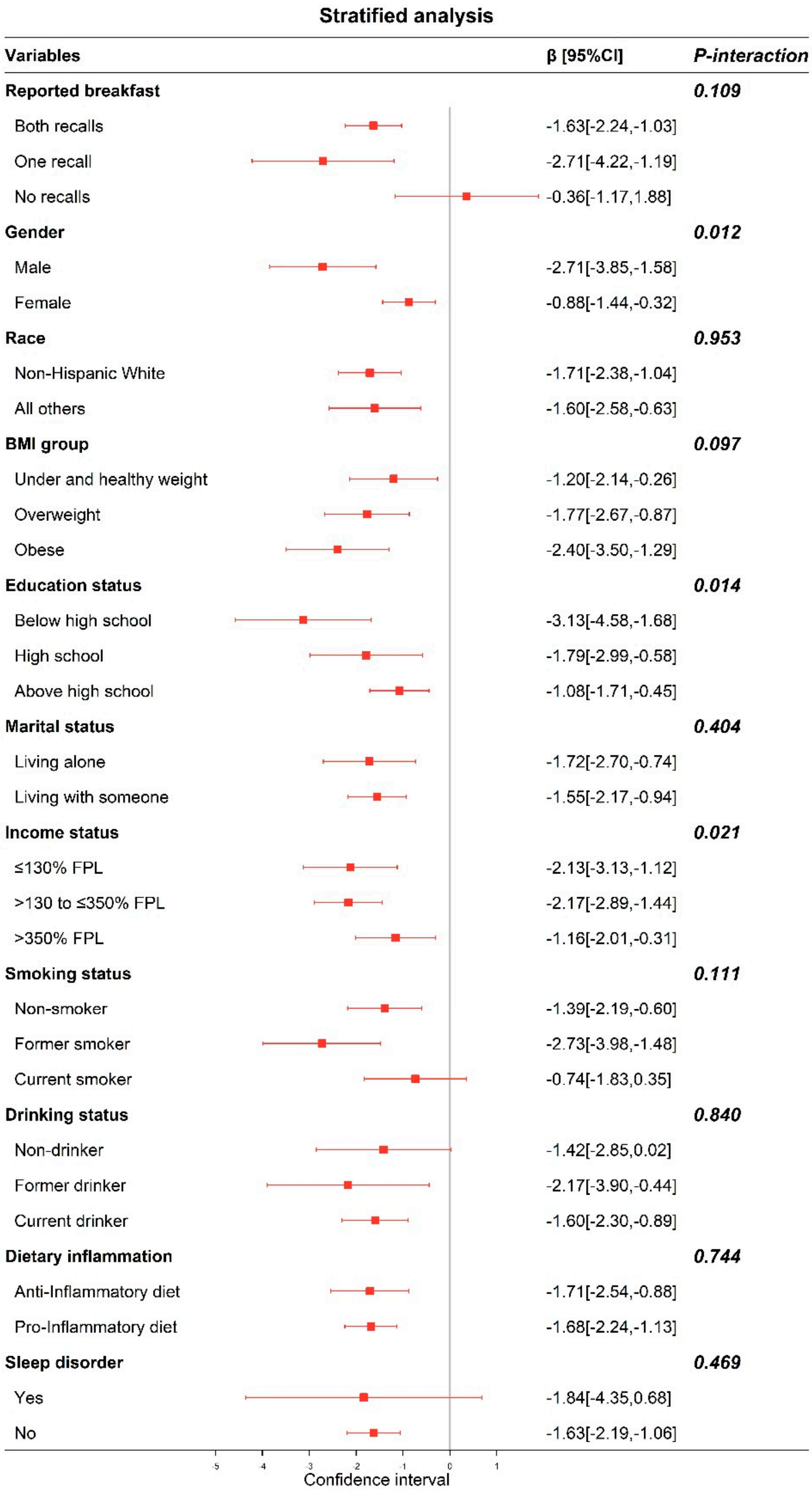

Figure S1 Forest plot of stratified analyses of the associations between physical activity and PhenoAgeAccel. Note: the control group was physically inactive. Adjusted for reported breakfast, gender, race, education status, marital status, income status, BMI, DII, energy intake, smoking status, drinking status, and sleep disorder. Bold indicates the value of the *P*-

interaction for the grouping variable.
